# Nucleus factory on cavitation bubble for amyloid *β* fibril

**DOI:** 10.1038/srep22015

**Published:** 2016-02-25

**Authors:** Kichitaro Nakajima, Hirotsugu Ogi, Kanta Adachi, Kentaro Noi, Masahiko Hirao, Hisashi Yagi, Yuji Goto

**Affiliations:** 1 Graduate School of Engineering Science, Osaka University, Toyonaka, Osaka 560-8531, Japan; 2 Institute of Molecular Embryology and Genetics, Kumamoto University, 2-2-1 Honjo, Chuo-ku, Kumamoto 860-0811, Japan; 3 Center for Reserch on Green Sustainable Chemistry, Tottori University, 4-101 Koyama-cho minami, Tottori, Tottori 680-8550, Japan; 4 Institute for Protein Research, Osaka University, Yamadaoka 3-2, Suita, Osaka 565-0871, Japan

## Abstract

Structural evolution from monomer to fibril of amyloid *β* peptide is related to pathogenic mechanism of Alzheimer disease, and its acceleration is a long-running problem in drug development. This study reveals that ultrasonic cavitation bubbles behave as catalysts for nucleation of the peptide: The nucleation reaction is highly dependent on frequency and pressure of acoustic wave, and we discover an optimum acoustical condition, at which the reaction-rate constant for nucleation is increased by three-orders-of magnitudes. A theoretical model is proposed for explaining highly frequency and pressure dependent nucleation reaction, where monomers are captured on the bubble surface during its growth and highly condensed by subsequent bubble collapse, so that they are transiently exposed to high temperatures. Thus, the dual effects of local condensation and local heating contribute to dramatically enhance the nucleation reaction. Our model consistently reproduces the frequency and pressure dependences, supporting its essential applicability.

Amyloid-*β* (A*β*) peptides form neurotoxic aggregates (i.e., amyloid fibrils and oligomers) to cause Alzheimer’s disease (AD)^1^. Formation of A*β* aggregates often takes a very long time, and this slowness prevents us from clarifying detail aggregation mechanism of A*β* peptides. For revealing the aggregation mechanism of A*β* peptide, it is necessary to establish an effective acceleration method. It is recently suggested that aggregation reactions of several amyloidogenic proteins can be accelerated with ultrasonic irradiation, including *β*2-microglobulin^2^,^3^, *α*-synuclein^4^, prion protein^5^, hen egg white lysozyme^6^,^7^, insulin^8^, and A*β*_1−40_^9^. The mechanism of ultrasonically accelerated aggregation, however, remains unexplained, and its clarification will allow to control the aggregation reaction of A*β* peptides ultrasonically.

Promotion of chemical reaction by ultrasonic wave has been studied in the field of sonochemistry, where cavitation bubbles generated by ultrasonic wave are deeply related to sonochemical reactions^10^,^11^,^12^. Sonochemical reaction is principally a decomposition reaction for organic substances^13^,^14^: Ultrasonic cavitation bubbles repeat growth and collapse, and significant temperature increase (up to thousands of kelvins) occurs at the collapse, which promotes the pyrolytic decomposition of the soluble substances near the hot spot and also produces free radical species from water that oxidize the substances^15^,^16^,^17^. Such a sonochemical reaction has been also used in the study of amyloid fibrils to fibrillate aggregates for obtaining the seeds^2^,^18^.

The ultrasonic acceleration of aggregation, however, means polymerization of monomers, being the opposite reaction to the decomposition reaction. Thus, it is challenging to figure out its mechanism. The transformation from monomers to fibril principally takes two steps^19^,^20^,^21^. First, monomers interact with each other to cause a nucleus. Second, the nucleus elongates by capturing free monomers. Thus, nucleation and elongation are principal reaction schemes, although various modified models are proposed^22^,^23^, including transient presence of the secondary nucleus^24^,^25^. Because ultrasonic irradiation to well developed fibrils causes their breakdown into shorter fibrils^26^, we expect that the fibril-elongation reaction will not be markedly accelerated by ultrasonic irradiation. Because it is indicated that the energy barrier for nucleation is much higher than that for fibril growth^27^,^28^, the ultrasonic irradiation should affect the nucleation phenomenon so that a dramatic increase in the fibrillation rate is made possible. We, therefore, focus on enhancement of the nucleation process for clarifying the acceleration mechanism.

Cavitation bubbles are generated by negative pressure of driving ultrasonic wave. They could stably oscillate in case of low acoustic pressures, but show the transient cavitation behavior under high pressures, where bubbles grow relatively slowly and collapse rapidly, causing extremely high temperature region near the bubbles, called hot spots. The hot spot temperature exceeds 10,000 K in the single-bubble collapse^29^, and remains still high (~5,000 K) even in the multi-bubble cases^30^. Because the hot-spot temperature is highly affected by frequency and pressure of the driving acoustic wave^31^, we investigate ultrasonically accelerated aggregation behavior with various frequencies and pressures systematically in order to clarify its mechanism, which has not been done because of difficulty of controlling the acoustic field inside sample tubes. We also investigated the reaction rate constant by adding microbeads in the sample solutions without ultrasonic wave to separately search the interface effect on the nucleation reaction.

To reproduce these experimental results theoretically, we propose an aggregation acceleration model based on the dual effects of local condensation and local heating accompanied with the bubble collapse: A*β* monomers will be adsorbed on the bubble surface during bubble growth because of the presence of hydrophobic residues, they are condensed when the bubble shrinks for collapse, and they are exposed to extremely high temperature field near the hot spot at the bubble collapse. We computed the time-averaged macroscopic rate constant for nucleation based on this model. Our calculation succeeded in reproducing the presence of the optimum frequency for nucleation and dramatic increase in its velocity rate constant with increase in acoustic pressure. We thus find that the ultrasonic cavitation bubble shows catalytic behavior for enhancing the nucleation reaction of A*β* peptide, acting as the nucleus factory.

## Results

### Determination of activation energy for nucleation

Throughout this study, the thioflavin-T (ThT) assay was used for evaluating the progress of the aggregation reaction. Because ThT molecules specifically bind to the cross-*β*-sheet structure, which is unit structure of A*β* fibril, and cause emission, the ThT assay has been widely adopted for monitoring the aggregation reaction of A*β* peptides^32^,^33^.

For determining the activation energy for nucleation in our sample solutions, we performed incubation experiments with various temperatures between 298 and 323 K. Sample tubes including 500 *μ*L of the 10 *μ*M A*β*_1−40_ solution were incubated at a set temperature for ~50 h, measuring its ThT level every a few hours. Five measurements were independently performed at each temperature. The detailed experimental results are shown in Fig. S1.

The ThT-fluorescence-intensity change was analyzed by the two-step (Finke-Watzky) model, where the aggregation reaction is simply divided into nucleation and growth processes^20^,^21^. Concentration of fibril [*F*] at time *t* can be written as following:


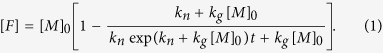


Here, 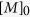 denotes the initial concentration of monomer. By fitting this two-step model, we extracted the reaction velocity constants for nucleation and fibril growth, 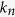 and 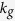, respectively. 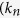 corresponds to the first-order rate constant in unit of 1/s, and 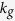 to the second-order rate constant in unit of L/mol·s.) From their temperature dependences, we determined the activation energies for nucleation and growth to be 61.9 and 7.8 kJ/mol, respectively, showing much higher energy barrier for nucleation as expected.

### Structure of amyloid fibril of A*β*_1−40_ peptide formed by ultrasonic irradiation

**Figure 1:**
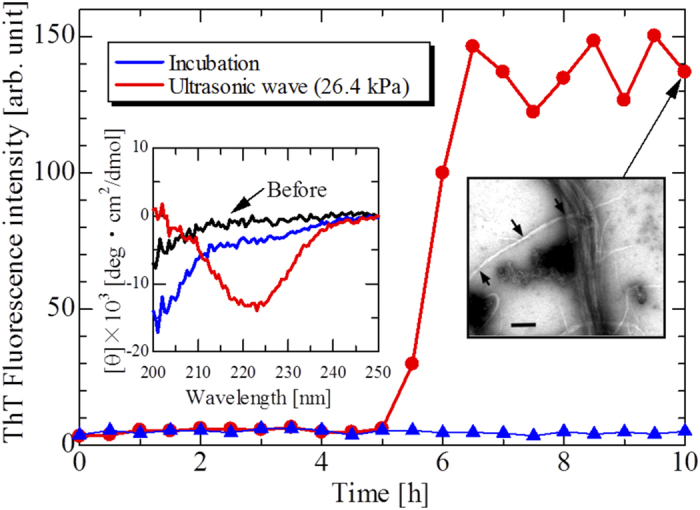
Aggregation reaction caused by ultrasonic irradiation. Time courses of ThT fluorescence intensity caused by ultrasonic wave with second-harmonics acoustic pressure of 26.4 kPa at fundamental frequency of 26 kHz and by incubation without ultrasonic wave. Insets show the CD spectra of initial monomer solution (black), incubated solution for 10 h (blue), and solution under ultrasonic irradiation (red); and a TEM image of aggregates formed by ultrasonic-wave irradiation for 10 h. The scale bar denotes 100 nm. The arrows in the TEM image indicate the minimum diameters of the twisted fibrils. Concentration of samples is 10 *μ*M.

Figure 1 demonstrates the accelerated behavior of the aggregation reaction with ultrasonic irradiation by home-developed experimental system shown in Fig. S2; the aggregation reaction was not observed for 10 h without ultrasonic wave, and its circular-dichroism (CD) spectrum (inset in Fig. 1) was nearly unchanged before the experiment, indicating that the secondary structure remains random coil without ultrasonic irradiation. However, by applying ultrasonic wave, the aggregation reaction was markedly accelerated (Fig. 1), clearly showing the minimum at 220 nm in the CD spectrum, indicating that amyloid fibrils with *β*-sheet-rich structures were formed with ultrasonic irradiation to the monomer solution. (The CD spectrum gives information related to the secondary structure of protein aggregates^34^.) Indeed, transmission-electron-microscopy (TEM) images of aggregates formed under ultrasonic irradiation displayed fibrils (inset in Fig. 1), which show similar morphology to that formed without ultrasonic irradiation: The amyloid fibrils with diameter of about 7 nm were found in brain tissues in AD patients^35^, and periodic-twist fibril structures were observed with periods about 80–250 nm without ultrasonic irradiation^35^,^36^,^37^. Our TEM images also show single fibrils with 6–10 nm diameters and periodic twist fibrils with ~150 nm period.

### Optimum frequency for the aggregation reaction

We performed ultrasonic-irradiation experiments with seven different frequencies (19, 29, 50, 71, 143, 208, and 239 kHz). For researching the frequency dependence of the aggregation reaction precisely, the acoustic pressure should be unchanged. The acoustic pressure was controlled by changing the input voltage for the Langevin-type ultrasonic transducer. It was measured by the handmade needle probe in each sample tube. We used the second-harmonics pressure as actual intensity of ultrasonic wave throughout this study because we found favorable correlation between the second harmonics pressure and the rate constant for nucleation as will be seen in the next section, which is not found in the case of fundamental acoustic pressure as shown in Fig. S3. The fundamental-mode ultrasonic-wave amplitude should be saturated or decreased as the acoustic power rises because of generation of cavitation bubbles, since acoustic negative pressure cannot be enhanced with bubbles. Cavitation bubbles, however, show highly nonlinear dynamics, producing strong harmonics components. Therefore, it should be more suitable for evaluating acoustic power inside the sample tube using harmonics amplitude. Actually, previous reports experimentally showed that the second-harmonics amplitude positively correlates with the input acoustic power^38^,^39^.

**Figure 2:**
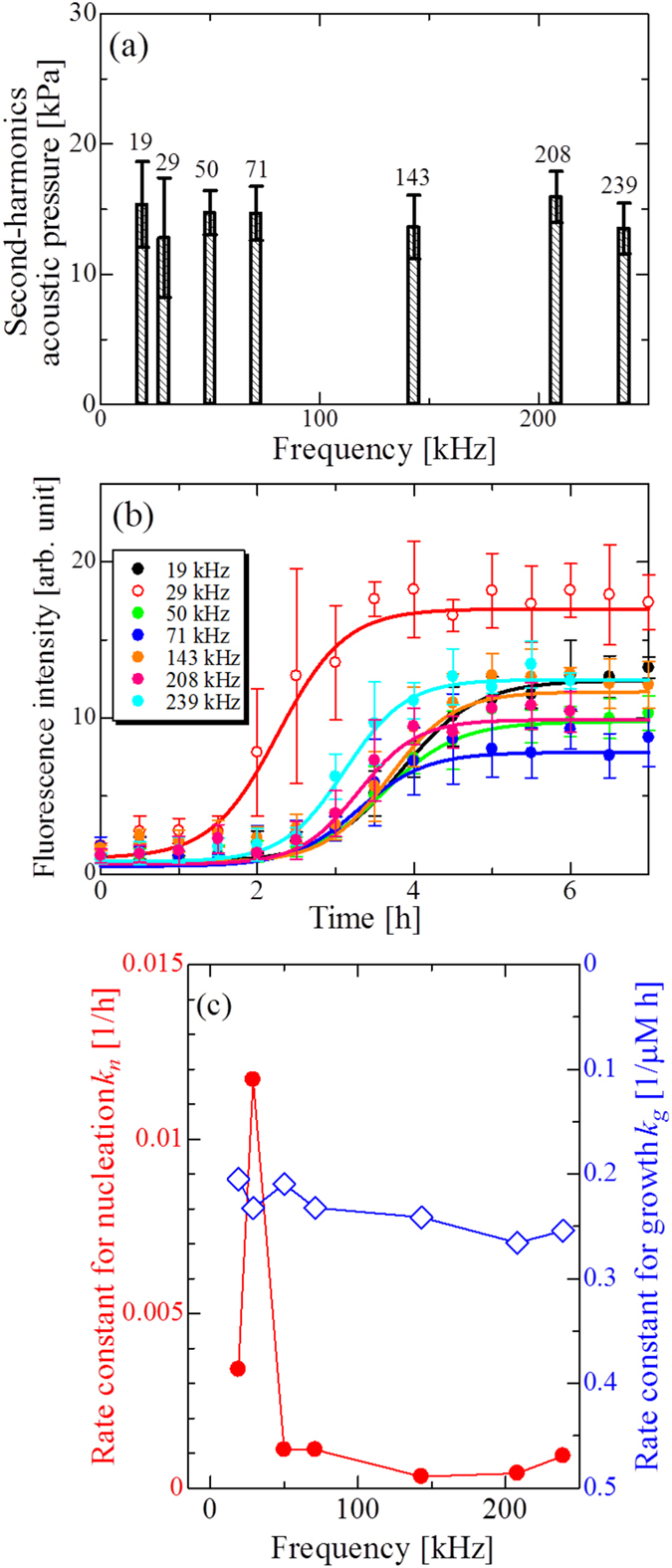
Frequency dependence of aggregation reaction of A*β* peptide. (**a**) Averaged second-harmonics acoustic pressure under ultrasonic wave at each frequency. Error bar denotes standard deviation in five sample tubes. Numbers are measured fundamental frequencies. (**b**) Time courses of ThT fluorescence intensity caused by ultrasonic wave with various frequencies (19, 29, 50, 71, 143, 208 and 239 kHz). Plots are averaged fluorescence intensity values of the five samples. Solid lines denote the fitted curves by the two-step model. The concentration of A*β* monomer solution was 10 *μ*M. (**c**) Frequency dependence of the rate constants for nucleation (solid circles), 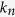, and for growth (open diamonds), 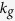.

The pressure at each frequency was controlled so as to achieve nearly the same second-harmonics-pressure value of ~15 kPa as shown in Fig. 2(a). (Waveforms inside the sample tubes and their corresponding Fourier spectra are shown in Figs S4 and S5.) The time course of the ThT fluorescence intensity at each frequency is shown in Fig. 2(b). Solid lines denote the fitted curves by the two-step model, from which we deduced the reaction velocity rate constants for nucleation 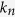 and for growth 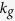. Figure 2(c) shows their frequency dependences. The best frequency is found at 29 kHz, at which the 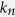 value is increased by a factor of 35 compared with the minimum value at 143 kHz. On the other hand, the 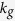 value is nearly independent of frequency.

From the experimental results, it is revealed that the optimal frequency exists in the ultrasonically induced nucleation reaction of A*β* peptides at 29 kHz. Frequency of ultrasonic wave strongly affects cavitation-bubble dynamics. In higher frequencies, the maximum size of cavitation bubble is restricted, and the temperature increase at the bubble collapse comes lower than at lower frequencies. Number of collapse per unit time and unit volume, however, increases at higher frequencies (Conventional sonochemical reactions are indeed enhanced as frequency increases in the frequency range used in this study^12^). The aggregation rate is therefore decided by balance between the temperature increase with bubble collapse and number of the collapse event per unit time, leading to an optimum frequency.

### Optimization of irradiation condition of ultrasonic wave

**Figure 3:**
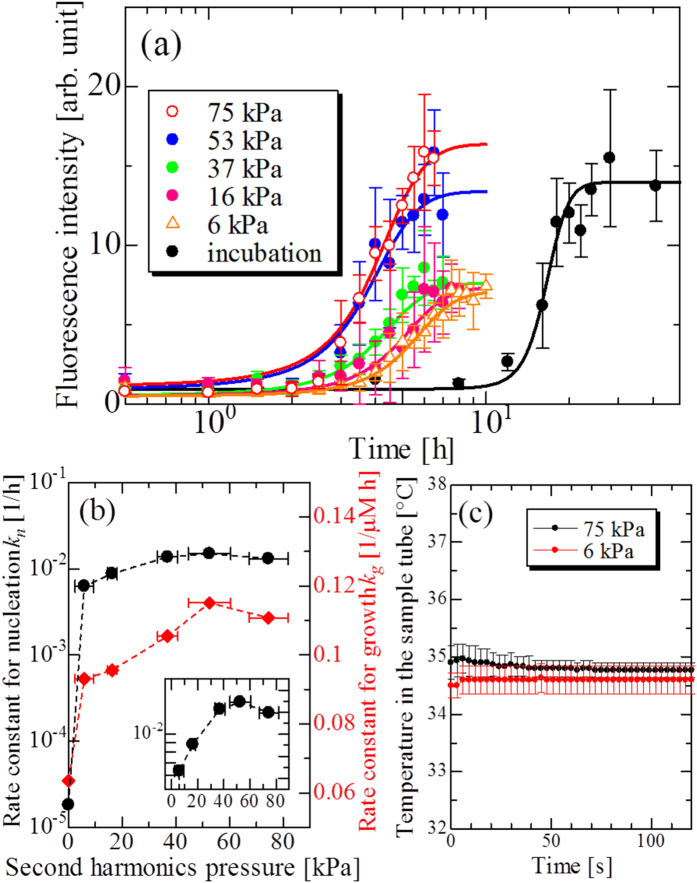
Pressure dependence of aggregation reaction of A*β* peptide. (**a**) Averaged time courses of ThT fluorescence intensity caused by various second-harmonics acoustic pressures. Solid lines denote fitted curves by the two-step model. The concentration of A*β* monomer solution was 10 *μ*M. (**b**) Relationship between the second-harmonics pressure of ultrasonic wave and the rate constants for nucleation (solid circles), 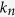, and for growth (solid diamonds), 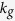. (**c**) Temperature change in the sample tube measured just after 1-min ultrasonic-wave irradiation with 75 and 6 kPa.

We further investigated the optimum condition of ultrasonic irradiation by changing the acoustic pressure at the best frequency of 29 kHz. Ultrasonic irradiation experiments with five acoustic pressures were performed. Figure 3(a) shows time course of ThT fluorescence intensity at each acoustic pressure, including incubation data without ultrasonic irradiation. Solid lines in Fig. 3(a) again show fitted curves by the two-step model. The waveforms inside the sample tubes under ultrasonic wave and their corresponding Fourier spectra are shown in Fig. S6.

Because the solution temperature significantly affects the aggregation reaction, we need to keep it to be constant to research intrinsic effect of ultrasonic wave on the aggregation reaction. Previous study on ultrasonically accelerated aggregation reaction actually revealed significant temperature increase in sample tubes during ultrasonic irradiation up to 50 °C^40^. We monitored temperature change just after the 1-min ultrasonic irradiation by inserting a needle thermocouple probe into sample tubes and confirmed that temperature increase there was less than 0.2 °C even at the maximum acoustic pressure as shown in Fig. 3(c).

Aggregation reaction comes faster with increasing the acoustic pressure. Surprisingly, 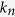 increases by a factor of ~1000 with presence of ultrasonic wave in comparison with quiescent condition, while 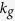 is less affected; it remains the same order (Fig. 3(b)). 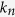 exhibits a positive correlation with the acoustic pressure between 0 to 53 kPa, and it slightly decreases from 53 to 75 kPa (inset in Fig. 3(b)). This depression indicates the decomposition reaction with a very large acoustic pressure, a typical sonochemical reaction.

### Effect of interface on reaction-rate constants

We investigated the effect of hydrophobic interface on the rate constant of nucleation reaction by adding microbeads into the sample solution to mimic the formation of ultrasonic cavitation bubbles. We added microbeads of 1 *μ*m in diameter with the volume fraction of 0.01%. (This volume fraction is the same as that used in our theoretical model below.) We used three kinds of microbeads made of silica, polystyrene with negative surface charge, and surface modified polystyrene with positive surface charge (see Materials and Methods). The result is shown in Fig. S7; the rate constants are increased on some level despite the surface charge of beads, indicating that the A*β* peptides should be captured on their surface because of their hydrophobicity rather than electrostatic interaction. However, the rate constant for nucleation is still much lower than the ultrasonically enhanced rate constant (Fig. S7(b)). This result reflects that the microbeads do not collapse in contrast to the case of ultrasonic cavitation bubbles.

## Discussion

### Aggregation acceleration model focused on cavitation-bubble dynamics

**Figure 4:**
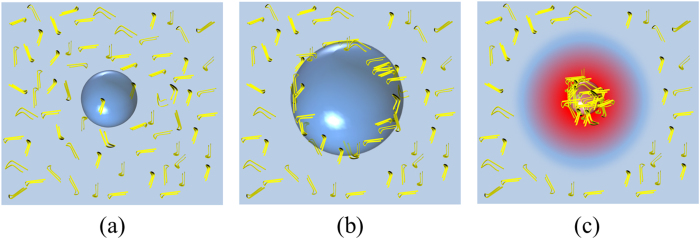
Aggregation acceleration model focused on cavitation-bubble dynamics. (**a**) Bubble is generated in solution by negative pressure of ultrasonic wave. (**b**) Bubble grows in the negative-pressure phase, during which A*β* monomers (yellow hairpin-shaped bars) are absorbed on bubble surface by hydrophobic interaction. (**c**) Bubble collapses at positive pressure of ultrasonic wave, leading to local condensation of A*β* monomers and temperature increase in and near the bubble.

The major experimental results are (i) the reaction-rate constant for nucleation favorably correlates with the second-harmonics acoustic pressure rather than the fundamental-mode pressure, (ii) the optimum frequency exists for the acceleration phenomenon, and (iii) it is remarkably increased by tuning the acoustic pressure at the best frequency. These observations highly indicate that the ultrasonic cavitation bubble should play a dominant role in the acceleration of nucleation process of A*β* peptide. We then attempt to reproduce these observations theoretically. Figure 4 illustrates our basic concept for the acceleration mechanism: Cavitation bubble is generated by ultrasonic irradiation to the A*β* monomer solution (Fig. 4(a)), and it expands during the negative-pressure phase. Because A*β* monomers include highly hydrophobic amino-acid residues^41^,^42^, they are absorbed to the bubble surface (liquid-gas interface) through the hydrophobic interaction (Fig. 4(b)). After that, the bubble collapses instantaneously with the positive pressure phase, and A*β* monomers absorbed on the bubble surface are attracted to the center, leading to condensation of monomers, which enhances the nucleation reaction. Besides, the bubble collapses in a short time of the order of 10 ns, inducing nearly adiabatic compression process. Temperature of gas inside the bubble then dramatically increases at the collapse, which reaches thousands kelvin degrees, and this makes temperature of surrounding solution much higher^30^. Based on the Arrhenius law, the nucleation reaction is accelerated by temperature increase around the bubble.

Therefore, the dual effects, “local condensation” and “local heating”, markedly enhance the nucleation reaction, which we consider as the principal mechanism of ultrasonically induced aggregation reaction: The bubbles work as catalysts for nucleation reaction of A*β* peptides. As indicated by Eisenberg and Jucker^43^, condensation and temperature increase both contribute to decrease the apparent energy barrier for amyloid formation, and it is important to recognize that these events are simultaneously induced by the bubble collapse. In following section, we propose a theoretical model to explain the dependences of the reaction rate constant on frequency and pressure of acoustic wave based on this aggregation acceleration model.

### Theoretical calculation

In this theoretical calculation, the transient breathing oscillation of bubble plays a critical role. We first calculated change in the bubble radius driven by ultrasonic wave based on the Keller-Miksis equation^31^ as shown in Supplementary Information. We then calculated temperature of gas inside bubble, assuming the adiabatic process. Examples of these calculations are shown in Figs S8(a–d). Sharp temperature increases around 40 and 80 *μ*s in Fig. S8 (b) correspond to the bubble-collapse events, at which temperature inside bubble reaches ~10,000 K; this is acceptable because it was experimentally shown that temperature inside bubble could reach near 10,000 K at bubble collapse^29^ for a single-bubble case, and this ultrahigh temperature field has been used for various chemical reactions^15^,^16^,^17^.

Next, we estimated temperature distribution around the hot spot by solving the sphere symmetrical thermal-diffusion equation: A sufficiently large spherical region (*R*_*B*_ = 1 mm), out of which temperature 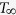 is unchanged, is considered, and the spherical hot spot is located at its center. The moment of bubble collapse, at which the bubble radius takes the minimum 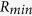 and the temperature increase takes the maximum 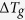, is defined as time *t* = 0. The hot spot gas region is replaced into the water without any dissipation, so that the temperature increase in the water core with radius 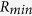, 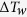 , is calculated by 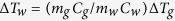 with the mass *m* and specific heat capasity *C*; subscripts *g* and *w* denote quantities of gas and water, respectively. With this step-like initial temperature distribution, we derived the time dependent temperature distribution 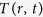 in water as follows (Detailed calculation process is given in Supplementary Information.):


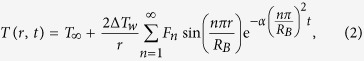



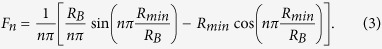


**Figure 5:**
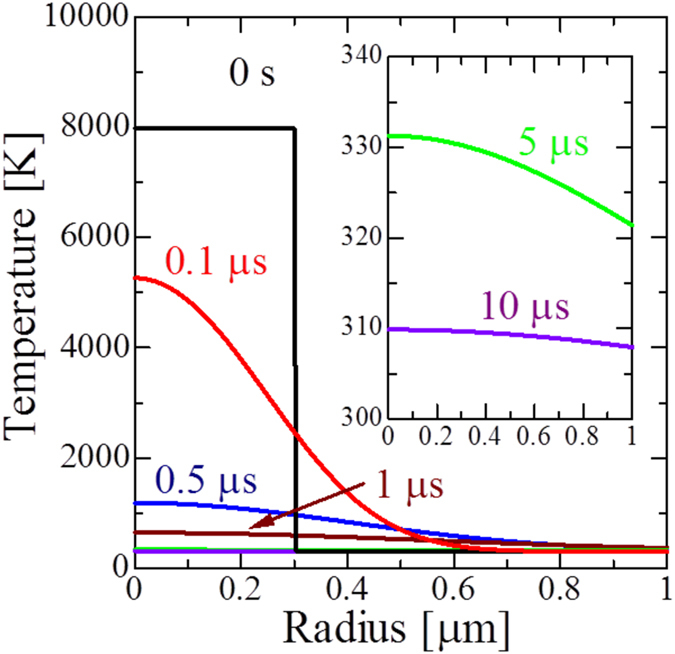
Evolution of temperature distribution near the hot spot. Used parameters are shown as following: *P*_*a*_ = 150 kPa, *f* = 29 kHz, *T*_∞_ = 310.15 K, *R*_*B*_ = 1 mm, *α* = 1.4 × 10^−7^ m^2^/s. Horizontal axis means the distance from the center.

Here, *α* denotes the heat diffusivity of water. An example of the calculation of thermal diffusion from the hot spot is shown in Fig. 5. Just after the bubble collapse, the core region immediately cools down to room temperature within ~10 *μ*s. The temperature affected zone is about 1 *μ*m from the core, indicating that this transient temperature increase affects only the peptides near the core and causes no influence on the others and the overall solution temperature as shown in Supplementary Information.

From thus calculated temperature profile, we intend to calculate the rate constant for nucleation 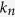 by assuming following hypotheses:

1. The number of captured peptides by the bubble equals to the number of peptides which existed in the volume of maximum-size bubble.
2. The volume fraction of bubble *ϕ* at its maximum is set to be constant 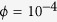 from the literature^44^, being independent of frequency and acoustic power.
3. All A*β* monomers attached on bubble surface are dragged at the core boundary at 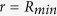 without being detached from the bubble surface. In other words, the fraction of A*β* monomers affected by the temperature increase at bubble collapse equals to the volume fraction of bubble *ϕ*.

The number of peptide excited into the nucleus state is estimated by the factor


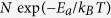
, where *N*, 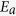, and 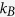 denote the total peptide number, activation energy for nucleation, and Boltzmann constant. We then obtain the time averaged rate constant for nucleation 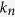 based on hypotheses above:


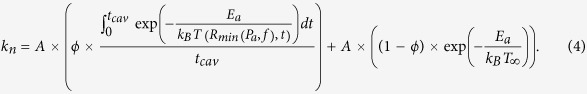


Here, *t*_*cav*_ and *A* denote the period of driving ultrasonic wave and the prefactor. The first term means the probability of nucleation of the peptides existing at the heat affected zone, and the second term is that of the non-heat-affected peptides, corresponding to the spontaneous nucleation. We used 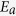 and *A* values obtained from the experiments at quiescent condition (Fig. S1(b)).

**Figure 6:**
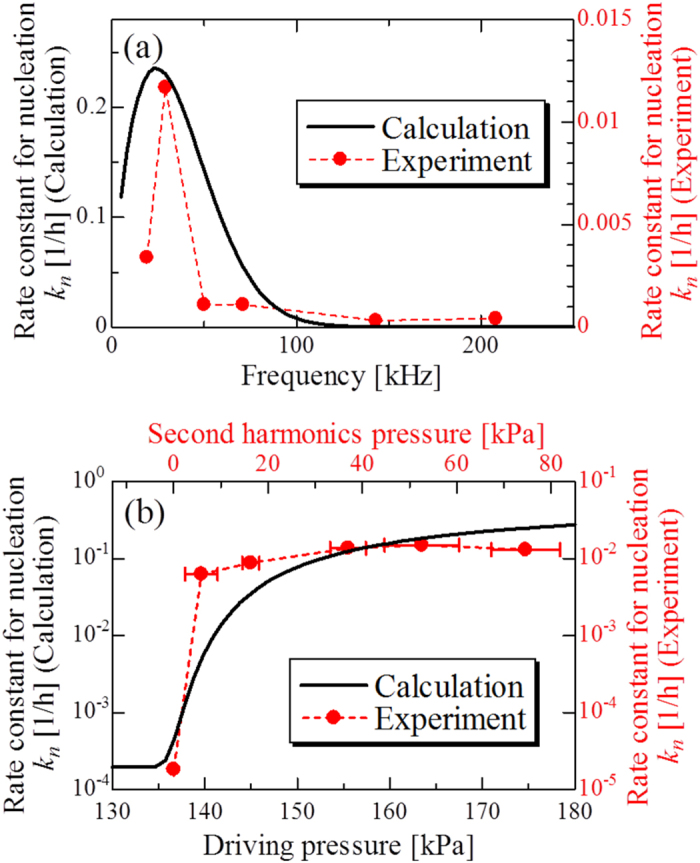
Calculation results of frequency and pressure dependence of *k*_*n*_. Comparisons between measured (red circles) and calculated (solid lines) (**a**) frequency and (**b**) pressure dependences of *k*_*n*_. For the calculation of the frequency dependence of 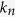, the driving pressure and equilibrium radius were set to *P*_*a*_ = 170 kPa and *R*_0_ = 2.0 *μ*m, respectively. For the calculation of the pressure dependence of *k*_*n*_, the frequency of ultrasonic wave and equilibrium radius were set to be *f* = 29 kHz and *R*_0_ = 2.0 *μ*m, respectively.

Calculation results of frequency and acoustic-pressure dependent 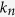 values are shown in Fig. 6. We succeeded in reproducing important tendencies of the aggregation reaction under ultrasonic field: There is a sharp peak in the frequency dependence, showing the best frequency near 30 kHz (Fig. 6(a)), and the rate constant increases drastically by many-orders-of magnitudes as driving pressure increases (Fig. 6(b)). These indicate the validity of our aggregation acceleration model.

The quantitative deviation of calculated 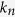 values from experiments is unavoidable, because there are some ambiguous parameters in the calculation, including the volume fraction of bubble (it may be dependent on acoustic power and frequency), the heat-transport loss from gas to water at collapse, and ambiguity in the driving acoustic pressure (*P*_*a*_ in Eq. (S1)), which cannot be experimentally obtained because cavitation bubbles modify the acoustic field significantly. Furthermore, some peptides will fail to follow the fast bubble-wall movement at the shrinking phase and they will be detached from the bubble surface away from the hot spot boundary, exposed to lower temperature, causing overestimation in deduced 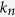. However, the important reaction characteristics remain regardless of ambiguities of these parameters.

We emphasize that our model expects negligible heating of the solution as shown in Supplementary Information; the overall-temperature increase of bulk solution is estimated to be less than 0.001 K, which hardly affects the reaction rate constant. Indeed, the measured solution-temperature increase caused by ultrasonic irradiation was less than 0.2 K as shown in Fig. 3(c). Therefore, the drastic increase in the reaction rate constant can not be explained by the bulk-solution-temperature increase by ultrasonic irradiation.

Importance of supersaturation for amyloid-fibril formation has been suggested^45^,^46^,^47^, where the fibril phase appears like crystallization. The dual effects (local condensation and local heating) proposed here could be interpreted as enhancement of supersaturation, because both densification and temperature raise accelerate the nucleation reaction of peptides, promoting the amyloid-fibril precipitation.

The aggregation experiment with microbeads (Fig. S7) supports the enhanced nucleation reaction by the local condensation with the presence of spherical interface. However, only this effect fails to explain our observations, because the reaction rate constant achieved by ultrasonic irradiation is much higher. Therefore, not only the local condensation on the surface, but also the local condensation by cavity collapse and the local heating are necessary for realizing the ultrafast nucleation reaction.

## Materials and Methods

### Preparation of materials

Lyophilized-powder A*β*_1−40_ peptides was purchased from Peptide Institute(Lot number: 4307-v). It was stored at −40 °C. First, A*β* was dissolved by dimethyl sulfoxide (DMSO). And then, sample solution was diluted to 10 *μ*M by a phosphate buffer saline (PBS) solution with pH = 7.4 containing 100 mM NaCl. The volume fraction of DMSO and the PBS solution is 1:4. The prepared A*β* sample was frozen at −40 °C again just before an aggregation experiment.

The ThT powder was dissolved to 5 *μ*M by glycine-NaOH buffer (the concentration of glycine was 50 mM and pH = 8.5) containing 100-mM NaCl. The ThT solution was stored with wrapping by aluminium foil to avoid fluorescence photobleaching. DMSO, PBS, NaCl, and ThT were purchased from Wako Pure Chemical Industries, Ltd. Glycine-NaOH buffer was purchased from Sigma Aldrich.

### TEM observation

The transmission electron microscopy (TEM) observation was performed using a system manufactured by HITACHI Corp. (H-7650). The acceleration voltage was set to 80 kV. Observation was performed by negative stain method with 2% acetate uranyl. First, sample solution is put on carbon-coated copper grid for 1 min for adsorption. And then, sample solution was stained by 2% uranyl acetate solution. Acquisition magnitude of images was 20,000.

### CD spectrum measurement

Circular dichroism (CD) spectrum measurement is a powerful tool for obtaining information related to the secondary structure of protein aggregates from difference of absorbance for right and left circular polarization. The *β*-strand structure which constitutes amyloid fibril shows negative absorption peak near 220 nm. Because DMSO has a large absorbance peak near 220 nm, A*β* was dissolved by 0.05% vol. ammonia solution for the CD-spectrum measurement. The measurement was performed with the instrument by JASCO J-820. The light path length of quartz cell was 1 mm. Cumulated number at the measurement was five times.

### Ultrasonic irradiation experiments

We developed an experimental system for ultrasonic irradiation experiments to A*β* solution. Its schematic illustration is shown in Fig. S2. The Langevin-type ultrasonic transducers with fundamental frequency of 40 and 28 kHz are manufactured by TAMURA Corporation (TBL4535D-40HN and TBL4535D-28HB). One of them is fixed to the bottom of the stainless-steel container by a clump, being detachable. Ultrasonic wave irradiates sample tubes containing the A*β* solution located near the water surface of the reaction container. The water in the reaction container was degassed by a degassing unit (KAIJO Corp., product No. 43103) for avoiding loss of acoustic pressure caused by scattering due to cavitation bubbles in the container. Temperature of degassed water is kept to be 37 °C by a temperature-controller unit in the buffer water tank. Acoustic pressure and frequency of ultrasonic wave are controlled by amplitude and frequency of input AC voltage from function generator (NF Electronic Instruments: WF1974), respectively. By setting several sample tubes in the circular pattern, as shown in Fig. S2(b), this experimental system allows us to perform ultrasonic irradiation experiments for five samples, simultaneously.

The water in the water bath was sufficiency degassed for 1 h. 1-min ultrasonic irradiation was performed, and then, the sample was incubated for 9 min. This 10-min sequence was repeated for ~10 h. Every 30 min, a 5-*μ*L A*β* sample was picked up from the sample tube and mixed with 50-*μ*L ThT solution in a quartz-crystal cell. The cell was set on the spectrophotofluorometer, manufactured by JASCO Corporation (Product number: FP-6200), where the ThT fluorescence intensity was measured with the excitation light of 450 nm and the detected light from 440 to 500 nm; the maximum fluorescence value was recorded as the fluorescence intensity at that sampling time.

In this study, the ThT molecules were added into the ultrasonic-irradiated samples, and they were not exposed to ultrasonic wave so as to avoid the influence of ultrasonication on the ThT activity. (Note that other previous studies^2^,^3^,^4^,^5^,^28^ used ThT-contained protein solutions for ultrasonic-irradiation experiments.)

To measure acoustic pressure inside the sample tube, we developed the needle-type probe. The PZT piezoelectric element was located on the top of the needle probe, and it transforms the acoustic-pressure waveform to voltage waveform. Transformed voltage waveform is imported to PC through a digitizer (National Instruments Corp.: USB-5133). We performed fast Fourier transform to the waveform to obtain the voltage value at each frequency. Obtained voltage value was converted into the acoustic pressure; its sensitivity was calibrated using the needle-type hydrophone (Toray Engineering Co. Ltd., NH8193), whose sensitivity is known. Calibration experiments were performed with various frequencies because the sensitivity of the probe depends on frequency. This hand-made probe was inserted to the sample tube, and the acoustic pressure was measured.

### Aggregation reaction with microbeads

We used three kinds of microbeads, silica beads, polystyrene beads, and surface modified polystyrene beads. All microbeads were purchased from Micromod (Lot number: 01-00-103 (silica), 43-00-103 (polystyrene), and 01-05-103 (surface modified polystyrene with trialkylammonium groups)). Silica and plane polystyrene beads possess negative surface charge and the surface-modified polystyrene beads show positive surface charge. We added the microbeads to the 10-*μ*M A*β* sample solutions which are identical with those used in the ultrasonic-irradiation experiments. The volume fraction of the beads was adjusted to be 0.01%, the same value as the bubble volume fraction in our theoretical model. The sample was incubated in a temperature-controlled chamber at 37 °C, and the ThT fluorescence intensity was measured every a few hours.

## Additional Information

**How to cite this article**: Nakajima, K. *et al.* Nucleus factory on cavitation bubble for amyloid *β* fibril. *Sci. Rep.* **6**, 22015; doi: 10.1038/srep22015 (2016).

## Supplementary Material

Supplementary Information
