# Supplementary material for: Nucleus factory on cavitation bubble for amyloid β fibril
Source: Sci Rep. 2016 Feb 25;6:22015. doi: 10.1038/srep22015 (PMC4766559; doi:10.1038/srep22015)
Supplement: Supplementary Information [file srep22015-s1.pdf]

Supporting information

Nucleus factory on cavitation bubble for amyloid β fibril

Kichitaro Nakajima,[1] Hirotsugu Ogi,[1, a)] Kanta Adachi,[1] Kentaro Noi,[2] Masahiko Hirao,[1] Hisashi Yagi,[3] and Yuji Goto[4]

[1)]Graduate School of Engineering Science, Osaka University, Toyonaka, Osaka 560-8531, Japan
[2)]Institute of Molecular Embryology and Genetics, Kumamoto University, 2-2-1 Honjo, Chuo-ku, Kumamoto 860-0811, Japan
[3)]Center for Reserch on Green Sustainable Chemistry, Tottori University, 4-101 Koyama-cho minami, Tottori, Tottori 680-8550, Japan
[4)]Institute for Protein Research, Osaka University, Yamadaoka 3-2, Suita, Osaka 565-0871, Japan

[a)]Electronic mail: ogi@me.es.osaka-u.ac.jp

1. Bubble dynamics calculations by the Keller-Miksis equation

To calculate bubble radius motion, we performed single-bubble simulation with the Keller-Miksis equation:

$$\left(1-\frac{\dot{R}}{c}\right)\rho R\ddot{R}+\frac{3}{2}\left(1-\frac{\dot{R}}{3c}\right)\rho\dot{R}^2=\left(1+\frac{\dot{R}}{c}\right)\left[p_g(t)-P_0-P(t)\right]+\frac{R}{c}\dot{p_g}-4\eta\frac{\dot{R}}{R}-\frac{2\sigma}{R}\,,\qquad (S1)$$

$$p_g(t)=\left(P_0+\frac{2\sigma}{R_0}\right)\left(\frac{R_0}{R(t)}\right)^{3\gamma}\,,\qquad (S2)$$

$$P(t)=P_a\sin\left(2\pi f\left(t+\frac{R}{c}\right)\right).\qquad (S3)$$

Here, $R, \dot{R}, \ddot{R}, R_0, c, \rho, \eta$ and σ denote radius, bubble-wall velocity and bubble-wall acceleration, sound velocity, equilibrium radius of bubble, density, viscosity and surface tension of water, respectively. $p_g(t)$, $P(t)$, P_a and f denote the gas pressure inside bubble, acoustic pressure of ultrasonic wave, the pressure amplitude, and frequency of ultrasonic wave, respectively. Because bubble collapse occurs instantaneously (~10 ns), the process can be nearly regarded as adiabatic compression process. In adiabatic process, relationship between bubble radius and temperature of gas inside bubble can be written as following:

$$T(t) = T_0 \left(\frac{R_0}{R(t)}\right)^{3(\gamma-1)} . \qquad (S4)$$

Here, γ denotes ratio of specific heat.

Calculation results of the bubble radius motion and temperature change of gas inside bubble are shown in Fig. S8.

2. Calculation of solution-temperature field around the hot core

For calculating temperature field generated by thermal diffusion from the hot spot, we consider a heat-affected region with radius R_B (=1 mm), assuming that the temperature outside this region ($r>R_B$) remains constant to be T_∞. When the bubble radius becomes minimum R_{min} at collapse, temperature of gas inside the bubble reaches the maximum. We set this moment to be time t=0. In the spherical coordinate system, we analytically derive temperature increase by thermal diffusion from the heat source with radius R_{min} and temperature $T_\infty + \Delta T_w$. The governing equation is given by $\frac{\partial T}{\partial t} - \alpha \frac{1}{r^2}\frac{\partial}{\partial r}\left(r^2 \frac{\partial T}{\partial r}\right) = 0$ or

$$\frac{\partial (rT)}{\partial t} - \alpha \frac{\partial^2 (rT)}{\partial r^2} = 0 . \qquad (S5)$$

Here, α denotes thermal diffusivity of water. Initial condition and boundary condition are written by

$$T(r,0) = T_\infty + \Delta T_w (0 \le r \le R_{min}) , \quad (S6)$$

$$T(r,0) = T_\infty \ (R_{min} < r < R_B), \qquad (S7)$$

$$T(0,t) \ne \infty, \qquad (S8)$$

$$T(R,t) = T_\infty. \qquad (S9)$$

Here, ΔT_w is the temperature increase of the hot water core, which is obtained by assuming that

temperature increase of gas inside bubble conducts to the water core with no dissipation: $\Delta T_w = \frac{m_g c_g}{m_w c_w}\Delta T_g$, where ΔT_g denotes the temperature increase of the gas at collapse.

Setting $T'(r,t) \equiv T(r,t) - T_\infty$ and introducing the variable transformation $w=rT'$, the thermal diffusion equation and initial and boundary conditions (Eqs.(S6)-(S9)) can be rewritten as

$$\frac{\partial w}{\partial t} - \alpha\frac{\partial^2 w}{\partial r^2} = 0\,, \qquad (S5)'$$

$$T'(r,0) = \Delta T_w \;\; (0 \le r \le R_{min}), \qquad (S6)'$$

$$T'(r,0) = 0 \; (R_{min} < r < R_B), \qquad (S7)'$$

$$T'(0,t) \neq \infty\,, \qquad (S8)'$$

$$T'(R_B,t) = 0. \qquad (S9)'$$

General solution of Eq.(S5)' is obtained by method of separation of variables:

$$w(r,t) = (A sin\lambda r + B cos\lambda r)e^{-\alpha\lambda^2 t}. \qquad (S10)$$

Here, A, B and λ are constants. Thus, T' can be written as

$$T'(r,t) = \left(A\frac{sin\lambda r}{r} + B\frac{cos\lambda r}{r}\right)e^{-\alpha\lambda^2 t}. \qquad (S11)$$

Because T' takes a finite value at r=0, B=0. Thus, we have $T' = A\frac{sin\lambda r}{r}e^{-\alpha\lambda^2 t}$. Because a single solution fails to satisfy all the condition equations, we adopt linear superposition of solutions and explored A_n to satisfy the conditions:

$$T'(r,t) = \sum_{n=1}^{\infty} A_n \frac{sin\lambda_n r}{r} e^{-\alpha{\lambda_n}^2 t}. \qquad (S12)$$

From the condition (S9)', $sin\lambda_n R_B = 0$, which leads to $\lambda_n = \frac{n\pi}{R_B}$ $(n = 1,2,3\cdot\cdot\cdot)$. Thus, Eq. (S12) can be rewritten as

$$T'(r,t) = \sum_{n=1}^{\infty} A_n \frac{\sin\left(n\pi\frac{r}{R_B}\right)}{r} e^{-\alpha\left(\frac{n\pi}{R_B}\right)^2 t}. \qquad (S13)$$

The initial condition, that is the step-like temperature distribution at t = 0, gives

$$T'(r,0) = \sum_{n=1}^{\infty} A_n \frac{\sin\left(n\pi\frac{r}{R_B}\right)}{r} = \begin{cases} \Delta T_w \; (0 \le r \le R_{min}) \\ 0 \quad (R_{min} \le r \le R_B) \end{cases}. \qquad (S14)$$

$$\Leftrightarrow \sum_{n=1}^{\infty} A_n \sin\left(n\pi\frac{r}{R_B}\right) = \begin{cases} r\Delta T_w \; (0 \le r \le R_{min}) \\ 0 \quad (R_{min} \le r \le R_B) \end{cases}. \qquad (S14)'$$

We multiply Eq. (S14)' by $\sin\left(m\pi \frac{r}{R_B}\right)$ and integrate it between r=0 and R_B:

$$\int_0^{R_B} \sum_{n=1}^{\infty} A_n \sin\left(n\pi \frac{r}{R_B}\right) \sin\left(m\pi \frac{r}{R_B}\right) dr = \Delta T_w \times \int_0^{R_{min}} r \sin\left(m\pi \frac{r}{R_B}\right) dr. \qquad \text{(S15)}$$

From $\int_0^{R_B} \sin\left(n\pi \frac{r}{R_B}\right) \sin\left(m\pi \frac{r}{R_B}\right) dr = \begin{cases} 0 \ \ (m \neq n) \\ \frac{R_B}{2} \ \ (m = n) \end{cases}$, Eq. (S15) can be rewritten as

$$A_n \times \frac{R_B}{2} = \Delta T_w \times \int_0^{R_{min}} r \sin\left(n\pi \frac{r}{R_B}\right) dr. \qquad \text{(S15)}'$$

By calculating integration of right hand of Eq. (S15)', A_n is obtained as following:

$$A_n = \frac{2\Delta T_w}{n\pi} \left\{ \frac{R_B}{n\pi} \sin\left(n\pi \frac{R_{min}}{R_B}\right) - R_{min} \cos\left(n\pi \frac{R_{min}}{R_B}\right) \right\}. \qquad \text{(S16)}$$

Then we obtain the temperature distribution around the bubble as following:

$$T(r,t) = T_{inf} + \frac{2\Delta T_w}{r} \sum_{n=1}^{\infty} F_n \sin\left(n\pi \frac{r}{R_B}\right) e^{-\alpha\left(\frac{n\pi}{R_B}\right)^2 t}, \qquad \text{(S17)}$$

where

$$F_n = \frac{1}{n\pi} \left\{ \frac{R_B}{n\pi} \sin\left(n\pi \frac{R_{min}}{R_B}\right) - R_{min} \cos\left(n\pi \frac{R_{min}}{R_B}\right) \right\}. \qquad \text{(S18)}$$

3. Estimation of overall solution temperature

For roughly estimating temperature increase of bulk solution caused by bubble collapse, we calculated temporal and spatial average of temperature distribution around the bubble (Eq. (S17), (S18)):

$$T_{ave} = \frac{\int_0^{R_{cav}} \int_0^{t_{cav}} T(r,t)\, dt\, dr}{R_{cav} \times t_{cav}} \qquad \text{(S19)}$$

Here, R_{cav} and t_{cav} denote the half distance between bubbles and period of ultrasonic wave, respectively. The volume fraction of bubble is reported to be ~10^{-4} from the literature: $\frac{V_{cav}}{V_{total}} = 10^{-4}$. The total volume of solution is 500 μL (=5×10^{-7} m^3) in our experiment. Thus, the total volume of cavitation bubbles is V_{cav}=5×10^{-11} m^3. For example, at the acoustic pressure of 150 kPa and frequency of 29 kHz, the maximum radius of the bubble is R_{max}= 53.29 μm, and the volume of one bubble, $V_{1\text{-}cav.}$, is $V_{1-cav.} = \frac{4\pi R_{max}^3}{3} = 6.34\times10^{-13}$ m^3. Thus, the number of cavitation bubble, $n_{cav.}$, is $n_{cav.} = \frac{V_{cav.}}{V_{1-cav.}} \approx 79$. By assuming that bubbles are dispersed homogeneously, the volume of sphere region affected by one bubble is $V_{total} \div 79 = 6.3\times10^{-9}$ m^3, and the radius, $R_{cav.}$, is

$R_{cav} = 1.1$ mm.

Using this value, we numerically computed the average temperature increase, $T_{ave.}$, based on Eq. (S19), and find that the overall temperature increase is less than 0.001 K; nearly unchanged. This is because the high temperature region is highly localized near the hot spot and exists within a very short time.

4. Supplementary Figures S1-S7

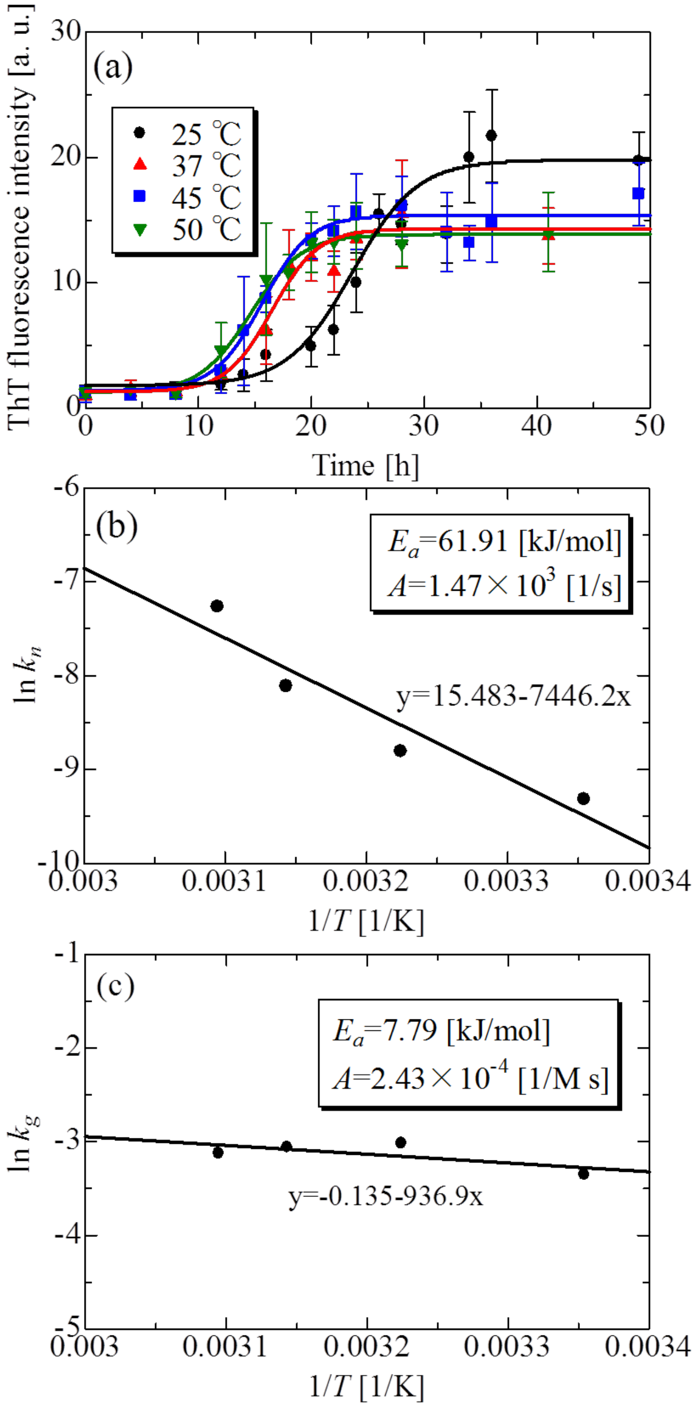

Fig.S1 Result of incubation experiments with various ambient temperatures (25, 37, 45 and 50 °C) and determination of activation energies for nucleation and growth. (a)Time course of ThT fluorescence intensity at each temperature. The solid lines denote fitted theory based on the two-step model. The Arrhenius plots for determining activation energies for (b) nucleation and (c) growth of A$\beta_{1\text{-}40}$ fibril, respectively.

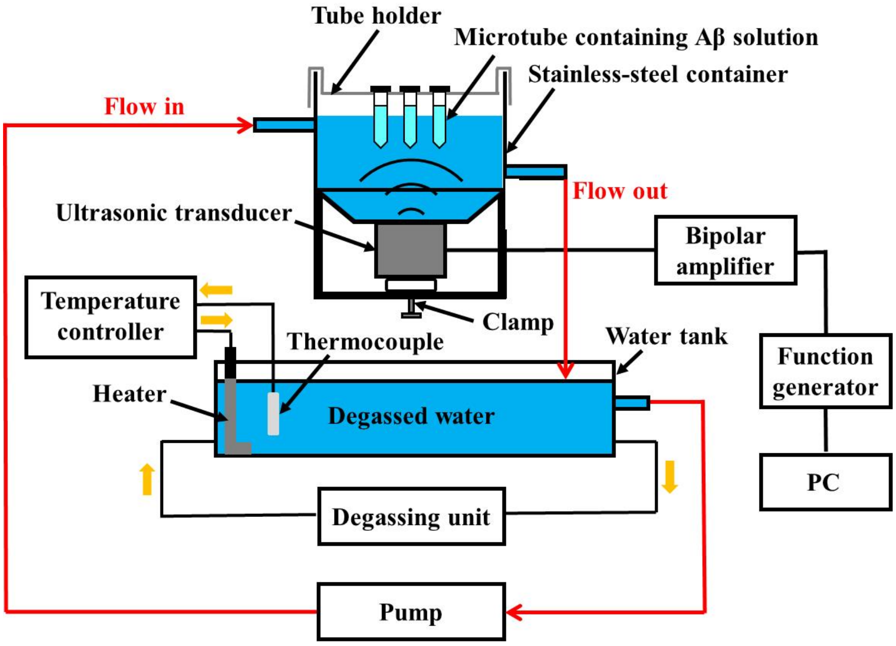

(a) Schematic view of an experimental system.

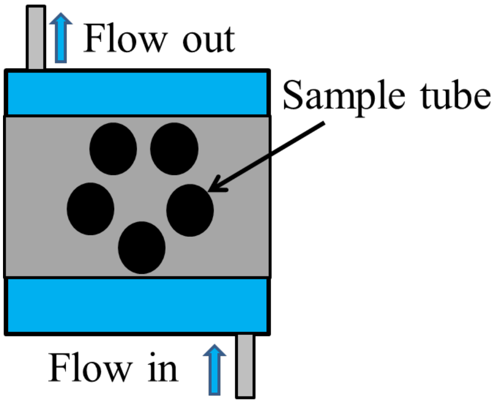

(b) Top view of the reaction container.

Fig.S2 (a) Schematic of the home-built experimental system for researching frequency and acoustic pressure dependences of aggregation reaction of A$\beta_{1\text{-}40}$ peptide. We used two Langevin type ultrasonic transducers with fundamental frequencies of 28 and 40 kHz; the former was used for frequencies below 200 kHz, and the latter for those beyond 200 kHz. One of them was strongly fixed to the bottom of the container by a clump. The water was degassed by the degassing unit and its temperature was kept at 37 °C by the temperature controlling system. (b) Top view of the reaction container, showing five sample tubes in a circular pattern. This measurement setup allows us to perform ultrasonic irradiation experiments on five sample solutions simultaneously with nearly the same irradiation condition.

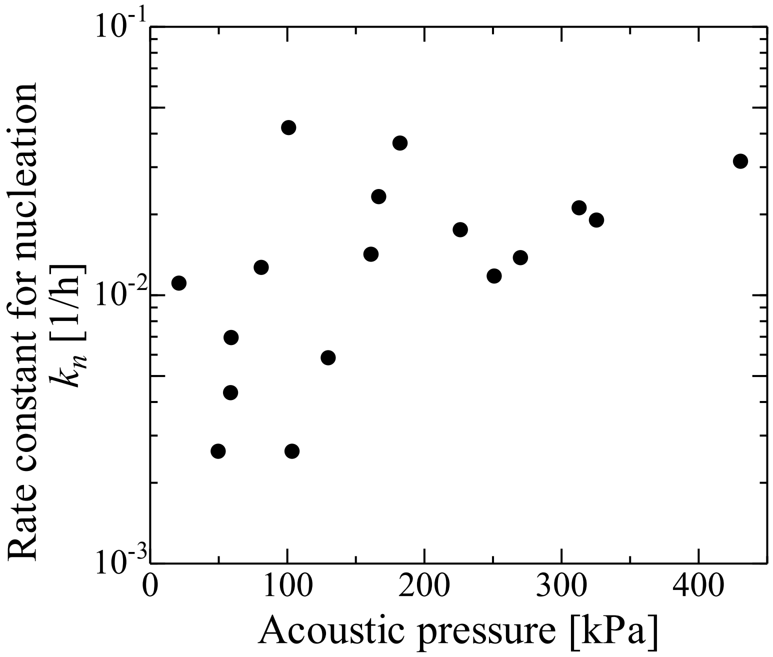

Fig. S3 Relationship between the rate constant for nucleation k_n and the acoustic pressure of the fundamental mode. No clear correlation is indicated between them.

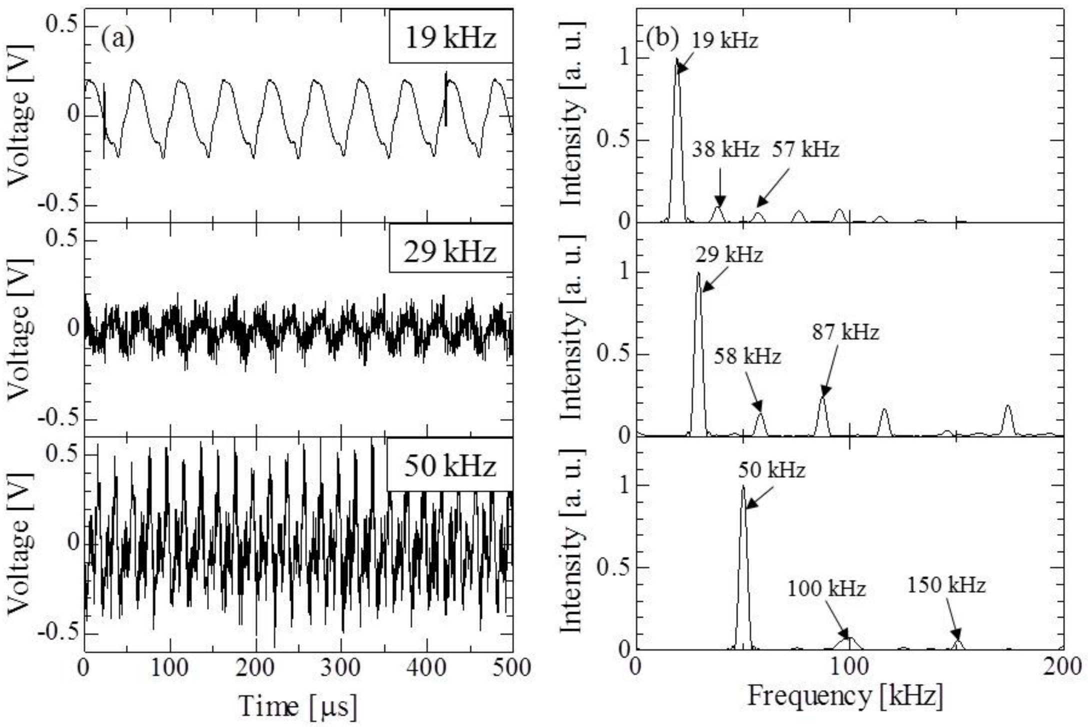

Fig.S4 (a) Representative acoustic waveforms observed in sample tube under ultrasonic irradiation with fundamental frequencies of 19, 29 and 50 kHz, and (b) their corresponding FFT spectra.

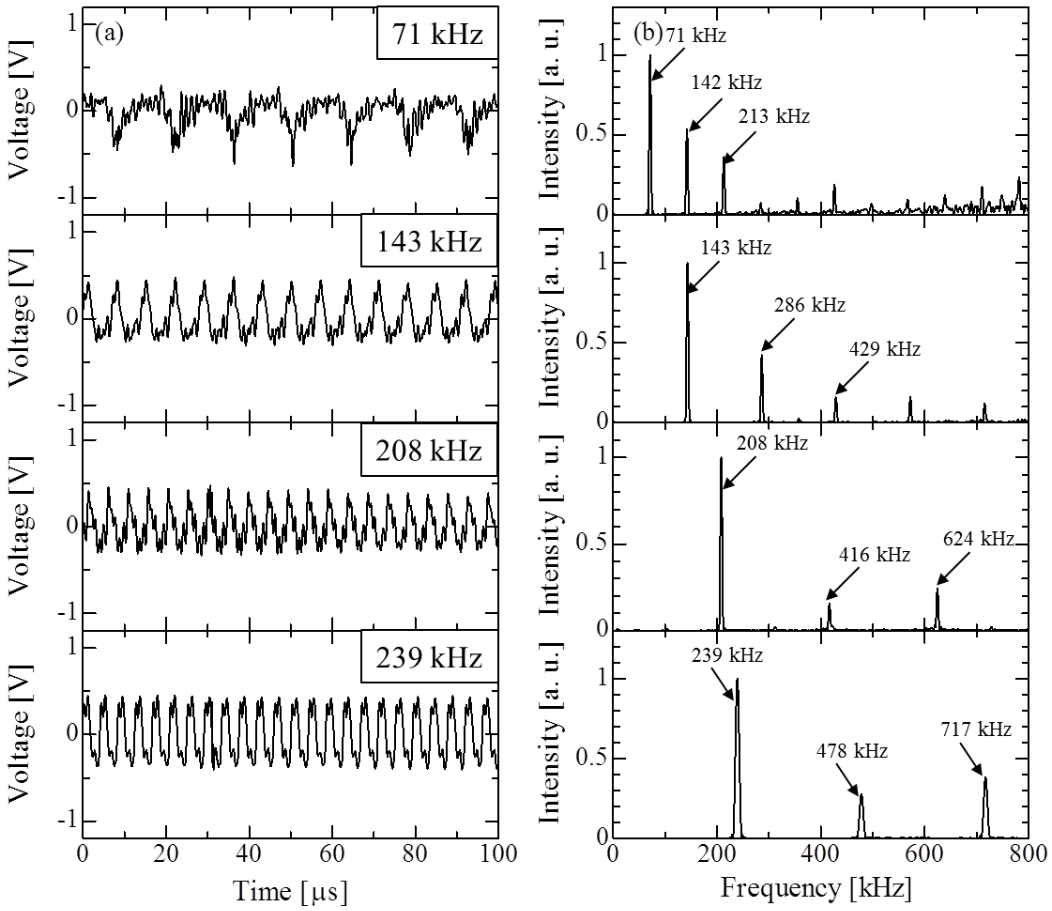

Fig.S5 (a) Representative acoustic waveforms observed in sample tube under ultrasonic irradiation with fundamental frequencies of 71, 143, 208 and 239 kHz, and (b) their corresponding FFT spectra.

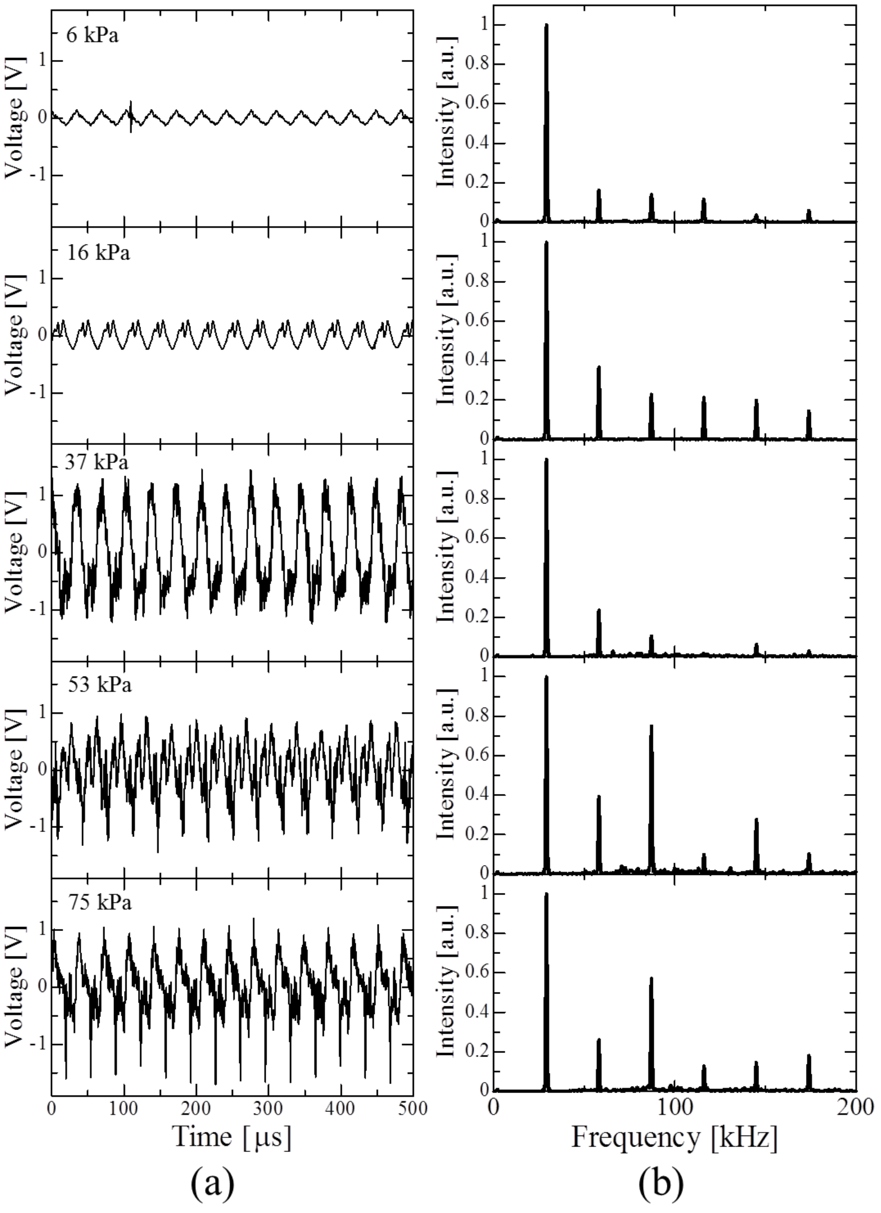

Fig.S6 (a) Representing waveforms and (b) their corresponding FFT spectra observed in experiments related to the acoustic-pressure dependence of Aβ aggregation reaction with fundamental frequency of 29 kHz. The averaged second-harmonics pressure values are shown in (a).

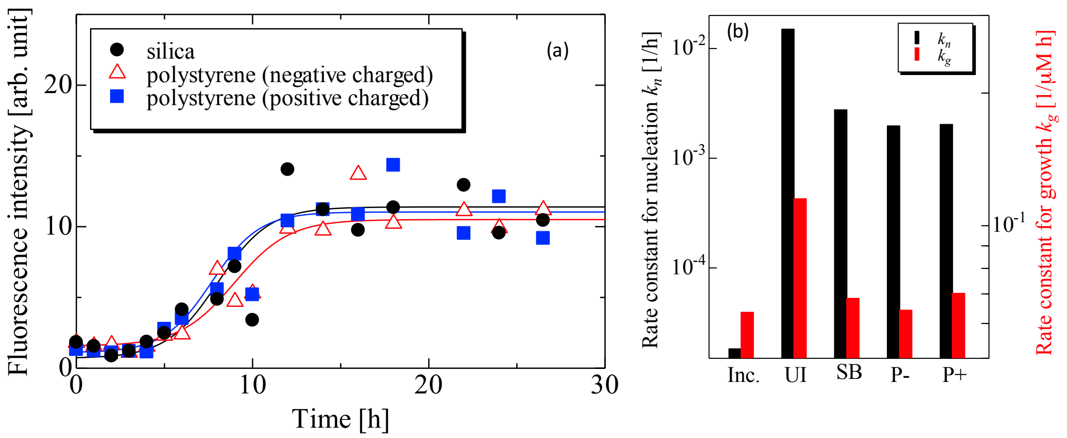

Fig.S7 (a) Evolution of ThT fluorescence intensity during incubation of Ab1-40 solutions involving silica beads, positively charged polystyrene beads, and negatively charged polystyrene beads. (b) Comparison of the reaction-rate constants among incubation (Inc.), optimized ultrasonic irradiation (UI), silica beads (SB), positively charged polystyrene beads (P+), and negatively charged polystyrene beads (P-).

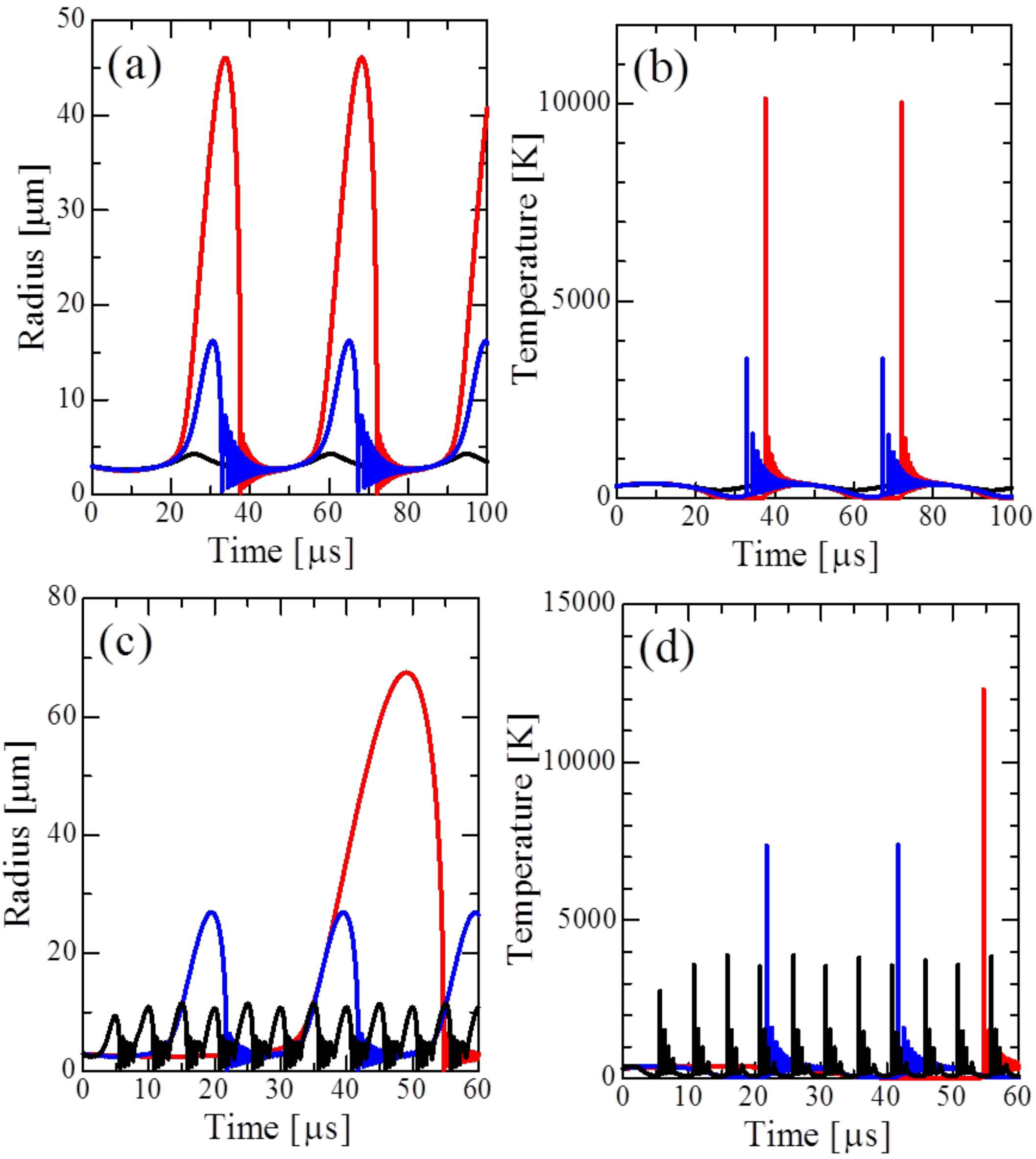

Fig. S8 Changes in bubble radius and temperature of gas inside bubble calculated by the Keller-Miksis equation. (a) and (b) show the changes of radius and inside temperature, respectively, with three acoustic pressures of P_a =100 kPa (black line), 125 kPa (blue line), and 150 kPa (red line) at frequency of 29 kHz. (c) and (d) show the changes of radius and inside temperature, respectively, with three different frequencies of 20 kHz (red line), 50 kHz (blue line), and 200 kHz (black line) under acoustic pressure of P_a =150 kPa. Used parameters are shown as following: P_0 = 101.3 kPa, R_0 = 2.0 μm, ρ = 993.9 kg / m^3, σ = 7.275×10^{-2} N / m, μ = 0.685×10^{-3} Pa s, c = 1533 m / s, and T_0 = 310.15 K.
